# Comparative efficacy of unilateral biportal endoscopy and micro-endoscopic discectomy in the treatment of degenerative lumbar spinal stenosis: a systematic review and meta-analysis

**DOI:** 10.1186/s13018-023-04322-2

**Published:** 2023-10-31

**Authors:** Hai Meng, Nan Su, Jisheng Lin, Qi Fei

**Affiliations:** Department of Orthopedics, Beijing Friendship Hospital, Capital Medical University, No 95, Yong’an Road, Xicheng District, Beijing, 100050 People’s Republic of China

**Keywords:** Degenerative lumbar spinal stenosis, Unilateral biportal endoscopy, Micro-endoscopic discectomy, Meta-analysis

## Abstract

**Background:**

Given the inconclusive literature on operative time, pain relief, functional outcomes, and complications, this meta-analysis aims to compare the efficacy of Unilateral Biportal Endoscopy (UBE) and Micro-Endoscopic Discectomy (MED) in treating Degenerative Lumbar Spinal Stenosis (DLSS).

**Methods:**

A thorough literature search was conducted in accordance with the PRISMA guidelines and based on the PICO framework. The study interrogated four primary databases—PubMed, Embase, Web of Science, and the Cochrane Library—on August 16, 2023, without time restrictions. The search employed a strategic selection of keywords and was devoid of language barriers. Studies were included based on strict criteria, such as the diagnosis, surgical intervention types, and specific outcome measures. Quality assessment was performed using the Newcastle–Ottawa Scale, and statistical analysis was executed through Stata version 17.

**Results:**

The meta-analysis incorporated 9 articles out of an initial yield of 1,136 potential studies. Considerable heterogeneity was observed in surgical duration, but no statistically significant difference was identified (MD = − 2.11, *P* = 0.56). For VAS scores assessing lumbar and leg pain, UBE was statistically superior to MED (MD = − 0.18, *P* = 0.013; MD = − 0.15, *P* = 0.006, respectively). ODI scores demonstrated no significant difference between the two surgical methods (MD = − 0.57, *P* = 0.26). UBE had a lower incidence of complications compared to those receiving MED (OR = 0.54, *P* = 0.036).

**Conclusions:**

UBE and MED exhibited comparable surgical durations and disability outcomes as measured by ODI. However, UBE demonstrated superior efficacy in alleviating lumbar and leg pain based on VAS scores. The findings present an intricate evaluation of the two surgical interventions for DLSS, lending valuable insights for clinical decision-making.

## Introduction

Degenerative lumbar spinal stenosis (DLSS) is a debilitating disorder characterized by the narrowing of the spinal canal, nerve root canals, lateral recesses, and intervertebral foramina. This pathological constriction commonly leads to symptomatic compression of nerve roots and/or cauda equina, manifesting in a range of clinical symptoms. DLSS is predominantly triggered by age-related degenerative changes in the lumbar vertebrae, with epidemiological studies indicating an increased prevalence correlating with advanced age [1]. One of the hallmark clinical symptoms of DLSS is neurogenic claudication. This condition presents as pain, cramps, or spasms in the buttocks, thighs, or calves during standing or walking, profoundly impacting motor functions and diminishing overall quality of life [2, 3]. Recent studies have underscored the severity of the disorder, revealing a general prevalence rate of 29%, which escalates to 47% among individuals aged 60 years and older [4]. While conservative treatments may offer varying degrees of symptomatic relief for patients with mild to moderate stenosis, surgical intervention becomes necessary for those with severe stenosis—particularly if symptoms persist or worsen after 3–6 months of conservative treatment [5]. Conventional surgical approaches for DLSS encompass laminectomy, lumbar spinal fusion, and interspinous process spacer insertion.

In recent years, there has been a monumental shift in the realm of spinal surgery, largely driven by technological advancements that have catalyzed the development and adoption of minimally invasive spine surgery (MISS). This transition has solidified MISS as a dominant paradigm in the surgical treatment of various spinal disorders, including DLSS. One of the most promising advancements within this surgical approach involves the utilization of endoscopic techniques. These encompass a range of methodologies, notably percutaneous endoscopy, micro-endoscopic discectomy (MED), and unilateral biportal endoscopy (UBE) [6, 7]. Endoscopic techniques offer several advantages over traditional open surgical procedures, such as reduced blood loss, decreased postoperative pain, shorter hospital stays, and quicker functional recovery. These benefits have made endoscopic techniques an attractive alternative, capturing considerable interest in medical academia and practice alike [8].

Although MED and UBE are widely used for treating DLSS, the clinical outcomes, prognostic factors, and safety profiles of UBE, a relatively novel technique, remain subjects of considerable debate and scrutiny. Numerous clinical trials have aimed to contrast the clinical efficacies of MED and UBE, yet a comprehensive systematic evaluation is still lacking. Consequently, this systematic review and meta-analysis aim to collate and analyze pertinent literature, comparing the effectiveness and safety of MED and UBE. By generating evidence-based insights, we seek to furnish clinicians with robust scientific data to guide decision-making in the treatment of DLSS.

## Materials and methods

### Search strategy

Throughout the conduct and subsequent dissemination of our meta-analytic findings, rigorous compliance was maintained with the PRISMA (Preferred Reporting Items for Systematic Reviews and Meta-Analyses) guidelines [9]. The structural organization of this meta-analysis was established based on the PICO (Patient, Intervention, Comparison, Outcome) paradigm, expounding the specific components as follows: Patient Population (P): Individuals diagnosed with DLSS. Intervention (I): UBE for the surgical treatment of DLSS. Comparison (C): MED as an alternative surgical intervention for DLSS. Outcome (O): Comparative efficacy in terms of operation time, visual analog scale (VAS) scores, Oswestry disability index (ODI) scores, complication rates, and any other relevant clinical outcomes.

Four authoritative electronic databases—PubMed, Embase, Web of Science, and the Cochrane Library—were systematically interrogated on August 16, 2023, without the imposition of a temporal boundary. The search algorithm incorporated a strategic selection of keywords including 'Degenerative Lumbar Spinal Stenosis', 'Unilateral Biportal Endoscopy', and 'Micro-Endoscopic Discectomy,' deliberately curated to encapsulate the extensive range of the PICO elements and to ensure an exhaustive compilation of germane studies for inclusion in this meta-analysis. Absence of linguistic restrictions further widened the scope of inquiry. Moreover, the reference indices of pertinent articles were meticulously scrutinized to identify any supplemental potential sources.

### Inclusion criteria and exclusion criteria

*Inclusion Criteria* 1) Studies involving patients diagnosed with single-segment lumbar spinal stenosis, where the diagnosis is confirmed through clinical symptoms, signs, and radiological examination, and where symptoms have not significantly improved despite standardized conservative treatment; 2) Studies where the interventions involve a direct comparison between UBE and MED; 3) Outcomes measured should include operative duration, postoperative VAS scores for back and leg pain, postoperative ODI scores, intraoperative blood loss, rate of postoperative complications, and extent of postoperative dual expansion.

*Exclusion criteria* 1) Patients with comorbid conditions such as lumbar spondylolisthesis, lumbar scoliosis, ankylosing spondylitis, spinal tumors, fractures, or neurological disorders; 2) Patients with a history of surgery on the same lumbar segment; 3) Studies with poor quality and lack of original data; 4) Non-clinical studies or duplicated studies.

### Data extraction

The data extraction protocol for this meta-analysis involves two assessors working independently to extract and cross-verify information. Should any discrepancies arise during this process, the reviewers will engage in a discussion to reconcile these differences, and if necessary, a third reviewer may be consulted for arbitration. Data to be extracted include authorship and year of the included studies, gender distribution (Male/Female), age of participants (in years), involved spinal segment, type of literature along with its quality score, duration of follow-up (in months), and observed outcome measures. In instances where the published reports lack essential data, outreach will be made to the authors of the original studies via email to request the missing, unpublished information. This comprehensive approach ensures systematic and rigorous data extraction, aligned with meta-analysis best practices.

### Quality assessment

In the forthcoming meta-analysis, the caliber of the included studies will be stringently appraised by a pair of autonomous reviewers utilizing the Newcastle–Ottawa Scale (NOS) as an evaluative metric [10]. The NOS is a widely accepted appraisal instrument consisting of nine criteria distributed over three pivotal domains: selection, comparability, and outcome assessment. This framework allows for a thorough scrutiny of potential bias within the incorporated studies. Subsequent to this exhaustive analysis, each study will be allocated a quality score on a scale spanning from 0 to 9. Interpretively, studies securing scores within the 0–3 range are categorized as low-quality research; those achieving scores between 4 and 6 are classified as moderate-quality research; and studies attaining scores in the 7–9 range are considered to epitomize high-quality scholarship.

### Statistical analyses

In our forthcoming meta-analysis, the evaluation of study heterogeneity will be carried out utilizing Chi-squared tests and will be quantitatively assessed through the *I*^2^ statistic. A condition of *I*^2^ values below 50% accompanied by a *P*-value equal to or greater than 0.10 signifies the absence of meaningful heterogeneity, leading to the application of a fixed-effect model for the amalgamation of effect sizes. Conversely, the manifestation of *I*^2^ values equal to or exceeding 50%, or a corresponding *P*-value below 0.10, is indicative of considerable heterogeneity. Under such circumstances, a random-effects model will be employed to synthesize the overall effect size. To assess the stability and integrity of our findings, sensitivity analyses will be executed, consisting of the sequential removal and re-computation of each study's impact on the global effect size. The funnel plot's symmetry will serve as a qualitative evaluation for the possible influence of publication bias on the study outcomes. Additionally, the quantitative appraisal for the existence of publication bias will be facilitated through Egger's linear regression test. All inferential statistics will be conducted as two-tailed tests, with a *P*-value of less than 0.05 constituting statistical significance. Data computations will be executed using Stata version 17, distributed by StataCorp, College Station, TX, USA.

### Evaluating the quality of evidence

To further complement the quality assessment of the included studies, we utilized the Grading of Recommendations Assessment, Development, and Evaluation (GRADE) scale. The GRADE approach evaluates the quality of evidence across studies for each primary outcome and facilitates the translation of evidence into practice. Evidence quality, for the purpose of this meta-analysis, was assessed across four grades: 1) High: Further research is very unlikely to change our confidence in the estimate of effect. 2) Moderate: Further research is likely to have an important impact on our confidence in the estimate of effect and may change the estimate. 3) Low: Further research is very likely to have an important impact on our confidence in the estimate of effect and is likely to change the estimate. 4) Very low: Any estimate of effect is very uncertain.

The quality grade was downgraded based on five factors: study limitations, inconsistency of results, indirectness of evidence, imprecision, and reporting biases. Conversely, upgrading considerations included a large magnitude of effect, plausible confounding that would decrease the demonstrated effect, and dose–response gradient.

## Results

### Search results and study selection

Upon conducting the initial database search, we identified 1,136 potentially relevant articles. Following the elimination of duplicate records and a meticulous screening process based on the predefined inclusion and exclusion criteria; we narrowed the selection down to 36 articles for further evaluation. Subsequently, 27 studies were excluded upon more detailed inspection, resulting in a total of 9 articles that met all the established criteria for inclusion in this meta-analysis [11–19].

Cumulatively, these nine articles represented a total sample size of 897 patients. Specifically, the sample sizes for the UBE and MED groups were rigorously extracted from each study and tabulated. The collected data revealed that the UBE group consisted of 417 patients, whereas the MED group had 480 patients. The distribution of these sample sizes across the studies is systematically detailed in Table 1, ensuring a comprehensive representation of the cohorts.

**Table 1:** Characteristics of studies included in the meta-analysis

Author	Publication year	Study type	Segment type	Age (years) UBE	Age (years) MED	Follow-up time (months)	Sample size of UBE	Sample size of MED
Aygun	2021	RCT	Single-segment	64.64 ± 10.09	65.01 ± 9.24	24	100	100
Ito	2021	R	Single-segment	66.3 ± 12.3	65.0 ± 11.1	6	42	139
Kim	2020	R	Single-segment	64.23 ± 5.26	66.20 ± 6.01	12	30	30
Min	2020	R	Single-segment	65.74 ± 10.52	66.74 ± 7.96	27.2	54	35
Park	2020	RCT	Single-segment	66.2 (41–80)	67.1 (45–79)	12	32	32
Choi	2019	R	Single-segment	65.4 ± 11.8	65.2 ± 12.0	6	35	30
Heo	2019	R	Single-segment	66.7 ± 9.4	63.4 ± 11.1	12	46	42
Kang	2019	RCT	Single-segment	65.1 ± 8.6	67.2 ± 9.5	6	32	30
Heo	2018	R	Single-segment	65.8 ± 8.9	63.6 ± 10.5	12	46	42

*RCT* Randomized controlled trial, *R* Retrospective

**Fig. 1:**
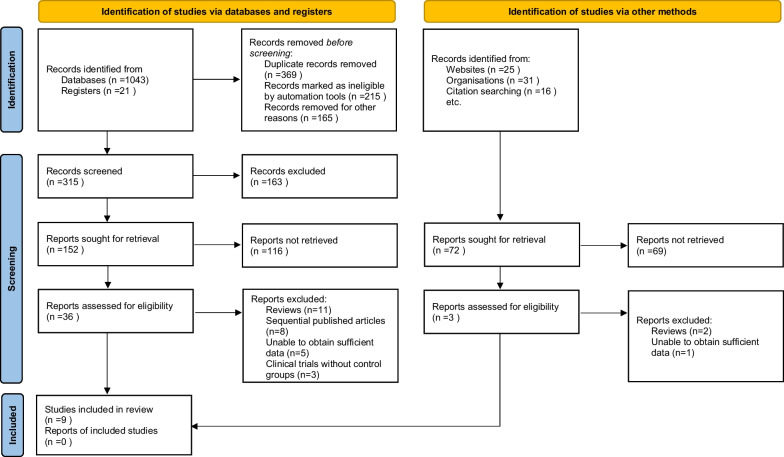
Selection process of included studies

The sequential stages of the literature selection process, along with the associated outcomes, are graphically illustrated in Fig. 1.

### Study characteristics

The meta-analysis encompasses a selection of studies that predominantly examine the effects of UBE and MED on single-segment spinal conditions. The studies included are a mixture of randomized controlled trials and retrospective studies, ranging in publication years from 2018 to 2021. The mean ages of participants treated with UBE and MED across the studies generally fall within the mid-60 s range, although there is considerable variability as indicated by standard deviations or age ranges. Follow-up periods for these studies range from 6 to 27.2 months (Table 1).

### Results of quality assessment

The evaluation of each study's methodological rigor was conducted using the NOS. Overall, the quality scores were distributed as follows: Two studies attained a score of 7, three studies received 8 points, and four studies garnered the highest score of 9. Notably, none of the included studies implemented blinding procedures or exhibited evidence of allocation concealment. Furthermore, no signs of funding bias were detected across the studies. Additionally, there were no instances of incomplete outcome data, premature termination bias, or imbalances at baseline among the evaluated studies. A comprehensive summary of the assessed risks of bias and their corresponding effect estimates can be found in Table 2.

**Table 2:** The quality assessment according to Newcastle–Ottawa scale of each cohort study

Study	Selection				Comparability	Outcome			Total score
	Representativeness of the exposed cohort	Selection of the non -exposed cohort	Ascertainment of exposure	Demonstration that outcome	Comparability of cohorts	Assessment of outcome	Was follow-up long enough	Adequacy of follow-up of cohorts	
Aygun		★	★	★	★★	★	★	★	8
Ito	★	★		★	★	★	★	★	7
Kim	★	★	★	★	★★	★		★	8
Min	★	★	★	★	★★	★	★	★	9
Park	★	★	★	★	★	★	★	★	8
Choi	★	★	★	★	★★	★	★	★	9
Heo	★	★		★	★	★	★	★	7
Kang	★	★	★	★	★★	★	★	★	9
Heo	★	★	★	★	★★	★	★	★	9

### Meta-analysis of surgical duration

**Fig. 2:**
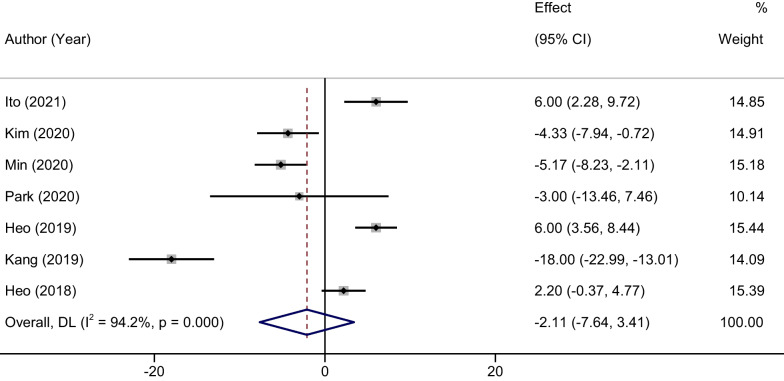
Forest plots of surgical duration

The heterogeneity assessment revealed substantial between-study variation (*P* < 0.001, *I*^2^ = 94.2%), thereby necessitating the employment of a random-effects model for the analysis. Despite the observed heterogeneity, the comparison between the two groups did not yield a statistically significant difference in surgical duration. The MD was − 2.11, with a 95% confidence interval ranging from − 7.64 to 3.41 (*P* = 0.56). A detailed representation of this finding is illustrated in Fig. 2.

### Meta-analysis of VAS scores assessing lumbar pain

**Fig. 3:**
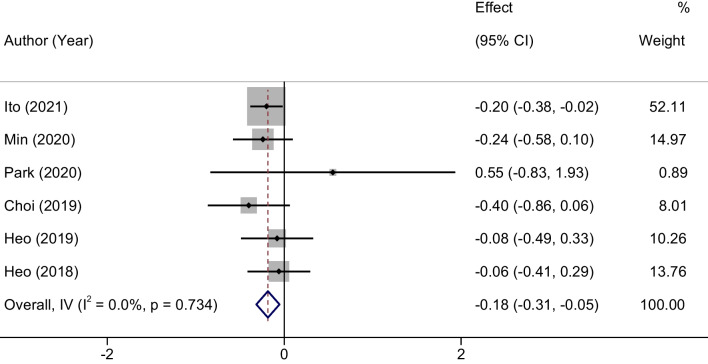
Forest plots of VAS scores assessing lumbar pain

In contrast to the results related to surgical duration, statistical homogeneity was observed among the studies for VAS scores assessing lumbar pain (*P* = 0.734, *I*^2^ = 0%). Consequently, a fixed-effects model was applied for this outcome measure. The analysis yielded a statistically significant MD in the final VAS scores between the groups, with an MD of − 0.18 and a 95% confidence interval ranging from − 0.31 to − 0.05 (*P* = 0.013). This suggests that the UBE group exhibited lower final VAS scores, indicative of better improvement in lumbar pain, compared to the MED group. These findings are graphically represented in Fig. 3.

### Meta-analysis of VAS scores assessing leg pain

**Fig. 4:**
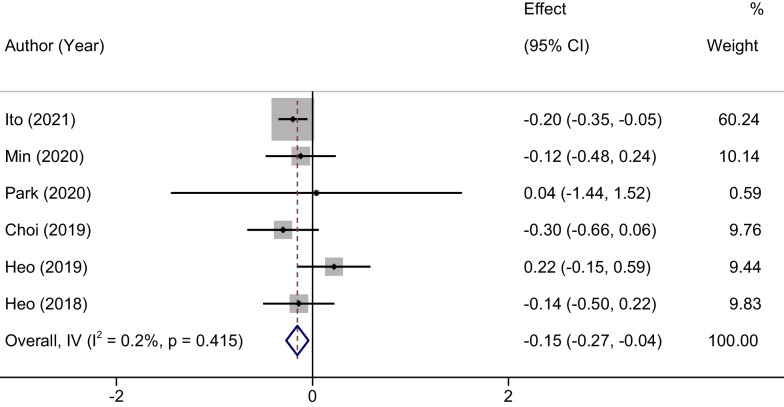
Forest plots of VAS scores assessing leg pain

Regarding the assessment of final leg pain using the Visual Analog Scale (VAS), statistical homogeneity was observed across the included studies (*P* = 0.415, *I*^2^ = 0.2%). Therefore, a fixed-effects model was employed for this particular outcome. The meta-analysis revealed a statistically significant MD in the final VAS scores for leg pain between the UBE and MED groups. Specifically, the MD was − 0.15, with a 95% confidence interval ranging from − 0.27 to − 0.04 (*P* = 0.006). This indicates that the UBE group exhibited superior amelioration of leg pain, as reflected by lower final VAS scores, compared to the MED group. These results are further elucidated in Fig. 4.

### Meta-analysis of Oswestry disability index Scores

**Fig. 5:**
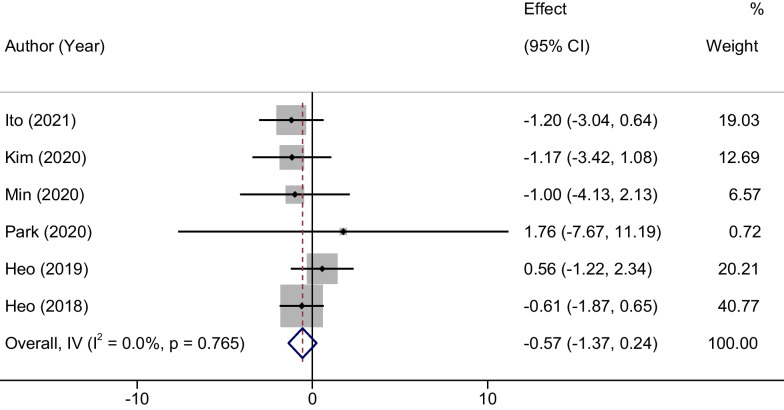
Forest plots of Oswestry disability index Scores

For the outcome measurement of the final Oswestry Disability Index (ODI) scores at the last follow-up, the meta-analysis demonstrated statistical homogeneity across the included studies (*P* = 0.765, *I*^2^ = 0%). Consequently, a fixed-effects model was implemented for this parameter. A statistical comparison between the UBE and MED groups revealed no significant difference in terms of their ODI scores; the MD was − 0.57 with a 95% CI ranging from − 1.37 to 0.24 (*P* = 0.26). This suggests that both treatment modalities, UBE and MED, resulted in comparable disability outcomes as measured by the ODI, which is illustrated in Fig. 5.

### Meta-analysis of the incidence of complications

**Fig. 6:**
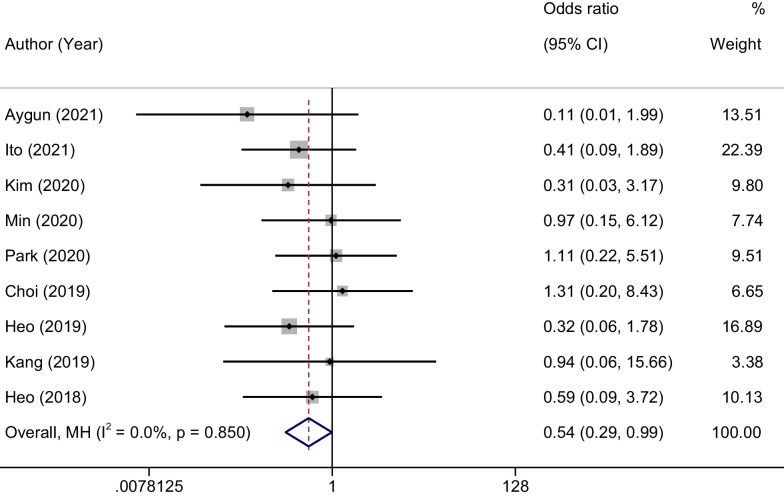
Forest plots of the incidence of complications

Our meta-analysis revealed no statistical heterogeneity among the incorporated studies (*P* = 0.850, *I*^2^ = 0%). Thus, a fixed-effects model was applied. The results indicated a statistically significant difference between the two groups in terms of complication rates. The odds ratio (OR) was 0.54 with a 95% CI of 0.29–0.99 (*P* = 0.036). These findings suggest that the UBE group had a lower incidence of complications compared to the MED group, as represented in Fig. 6.

### Sensitivity analysis of surgical duration

**Fig. 7:**
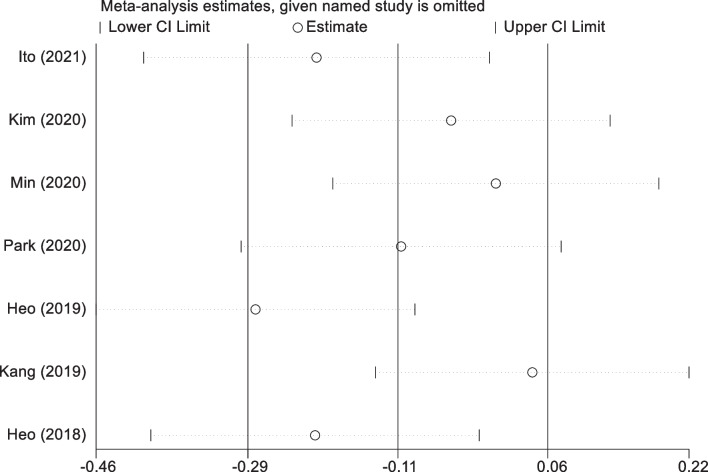
Sensitivity analysis of surgical duration

Owing to the marked heterogeneity identified among the studies incorporated into the meta-analysis, we executed a sensitivity analysis to scrutinize the resilience and dependability of the aggregated outcomes. This methodological step involved the iterative omission of each study, followed by the re-computation of the composite effect sizes based on the remaining studies. The meticulous sensitivity analysis affirmed that the aggregate results were consistently stable and robust, irrespective of the exclusion of any singular study from the analysis. Such findings signify that the influence exerted by any individual study did not disproportionately sway the comprehensive results, thereby augmenting the credibility of our meta-analytical conclusions (Fig. 7).

### Publication bias

**Fig. 8:**
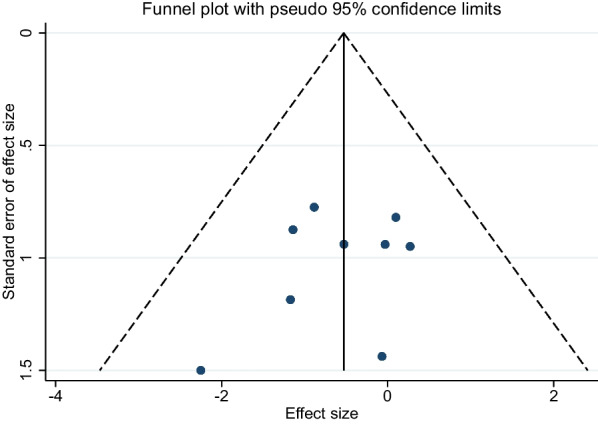
Funnel plot for publication bias in all included studies

The construction of funnel plots with the data from the included studies exhibited a symmetrical pattern, indicating an absence of substantial publication bias (Fig. 8). Further quantification using Egger's linear regression test revealed no discernible publication bias across varying variables, with all *p*-values exceeding 0.05. This additional validation serves to fortify the robustness and reliability of the findings generated by the meta-analysis.

### Certainty of evidence using the GRADE system

For a comprehensive understanding of the quality of evidence underpinning our findings, we assessed the certainty of evidence using the GRADE system. Here is a brief summary: 1) Operative Duration: The quality of evidence for this outcome was rated as "Moderate" due to inconsistency arising from substantial heterogeneity among studies. 2) Postoperative VAS Scores (Back Pain): The evidence for lumbar pain improvement was rated as "High", reflecting consistent findings across the studies without significant limitations. 3) Postoperative VAS Scores (Leg Pain): The evidence supporting the reduction in leg pain was rated as "High", indicative of consistent and robust results across the studies. 4) Postoperative ODI Scores: The evidence quality for this outcome was rated as "Moderate" because, despite consistent findings, there were concerns regarding potential biases in some included studies. 5) Intraoperative Blood Loss: This outcome was rated as "Moderate" due to possible biases within the analyzed studies.

This outcome achieved a "High" rating given the consistent findings across the studies, reflecting solid evidence for this measure. The evidence for this outcome was rated as "Low" due to indirectness, as few studies addressed this particular measure. The details of the certainty of evidence for each outcome, encompassing the risk of bias, inconsistency, indirectness, imprecision, and publication bias are elaborated in Table 3.

**Table 3:** Summary of certainty of evidence assessment for each outcome using the GRADE system

Outcome	Certainty of evidence	Risk of bias	Inconsistency	Indirectness	Imprecision	Publication bias
Operative duration	Moderate	Low	Moderate	Low	Low	Low
Postoperative VAS scores (Back Pain)	High	Low	Low	Low	Low	Low
Postoperative VAS scores (Leg Pain)	High	Low	Low	Low	Low	Low
Postoperative ODI scores	Moderate	Low	High	Low	Moderate	Low
Intraoperative blood loss	Moderate	Moderate	Moderate	Low	Moderate	Low

## Discussion

Micro-endoscopic discectomy and unilateral biportal endoscopy represent advanced surgical approaches in the field of spinal surgery, each with unique merits. MED is essentially a refinement of conventional surgical methodologies, enhanced by endoscopic technology [20]. It excels in minimizing surgical incisions, thereby reducing risks related to wound infections and aesthetic scarring. This benefit is particularly vital for patients who are more susceptible to wound-related complications. Additionally, MED's emphasis on tissue preservation drastically minimizes trauma to muscles and ligaments, thereby safeguarding the structural integrity of the spine. This minimal invasiveness fast-tracks postoperative recovery, including a quicker initiation of rehabilitation protocols, reduced lumbar pain, and shorter hospital stays—factors which cumulatively enhance patient experience and may also contribute to lower healthcare expenditure [21, 22].

UBE, although newer, has rapidly captured global interest due to its unique bilateral port system. The system, equipped with specialized arthroscopes, offers surgeons enhanced visibility during operations [23]. This unprecedented visual clarity facilitates precise surgical interventions, significantly elevating the efficacy of spinal decompression procedures. Moreover, the compatibility of UBE with standard surgical instruments makes it more accessible to surgeons experienced in traditional spinal procedures. One of its crowning features, however, is its unmatched adaptability. Unlike MED, UBE allows for decompression on both the ipsilateral and contralateral sides, a capability that is invaluable in addressing complex or bilateral spinal pathologies without resorting to multi-segmental decompression [24].

The inclusion of nine studies, of which three are high-quality RCTs, strengthens the internal validity of this meta-analysis, lending a higher degree of confidence to our findings. All studies targeted single-segment DLSS patients, thus narrowing the focus to a clinically relevant subset of patients who typically represent the majority of cases in many clinical settings. Our results indicate a nuanced interplay between the efficacy and safety profiles of MED and UBE, both of which are minimally invasive approaches for treating DLSS. They achieve similar operative times and ODI outcomes, signifying that neither technique has a substantial advantage over the other in these aspects. This is particularly interesting because it suggests that the choice between MED and UBE may ultimately depend on other, more specialized factors that could vary according to the individual needs of patients and the expertise of the surgical team. Our findings present a somewhat paradoxical situation: MED, while having a higher safety profile indicated by fewer complications, is less effective in postoperative pain control, as measured by VAS scores. On the other hand, UBE shows significant advantages in VAS outcomes but has a less favorable complication profile. This dichotomy may impact clinical decision-making. For example, in a patient population that is more vulnerable to complications—perhaps due to age or comorbid conditions—MED may be more appropriate. Conversely, for patients whose primary concern is postoperative pain, UBE may be the more suitable option.

Both our study and Zhou et al. [25] converge on the superiority of UBE over alternative methods in treating lumbar spinal stenosis. Similarities include UBE's efficacy in operation time and pain scores. However, our work uniquely emphasizes its advantage in lumbar and leg pain relief using VAS scores, while Zhou et al. highlight additional metrics like hospital stays and EuroQol 5-Dimension questionnaire. Importantly, our comprehensive search strategy, encompassing more databases until August 2023 and rigorous adherence to PRISMA and PICO frameworks, bolsters the robustness of our findings, offering a more updated and intricate evaluation for clinical decision-making. Our study rigorously analyzes the efficacy of surgical interventions for DLSS, focusing on pain relief, functional outcomes, and operative time. In contrast, Chen et al. [26] delve into the genomic landscape of Adrenocortical carcinoma (ACC), targeting metabolic gene signatures for prognostic evaluation. These distinct studies underscore different clinical challenges, rendering direct comparisons non-applicable, and emphasizing the breadth of ongoing medical research.

Despite the innovative contributions of both MED and UBE in spinal surgery, several limitations warrant caution in the interpretation and generalization of their benefits. First, the predominance of short-term studies for both techniques raises questions about their long-term efficacy and sustainability, particularly in patient populations with complex spinal pathologies or comorbidities. Second, the majority of existing research has primarily been conducted using single-center trials with relatively small sample sizes, limiting the robustness and external validity of the results. Finally, the comparative analyses between MED and UBE often lack a standardized set of outcome measures, making it challenging to draw definitive conclusions regarding their relative advantages and disadvantages in specific clinical scenarios.

## Conclusions

In conclusion, both MED and UBE are effective approaches for treating degenerative lumbar spinal stenosis. MED has the advantage of lower complication rates, while UBE excels in improving postoperative lumbar and leg pain, as measured by VAS scores. Therefore, a comprehensive consideration should be given when choosing between these surgical options to optimize patient outcomes.

## Data Availability

The datasets used and/or analyzed during the present study are available from the corresponding author on reasonable request.
